# Dietary energy restriction reduces high-fat diet-enhanced metastasis of Lewis lung carcinoma in mice

**DOI:** 10.18632/oncotarget.11598

**Published:** 2016-08-25

**Authors:** Sneha Sundaram, Lin Yan

**Affiliations:** ^1^ U.S. Department of Agriculture, Agricultural Research Service, Grand Forks Human Nutrition Research Center, Grand Forks, ND 58202, USA

**Keywords:** energy restriction, high-fat diet, Lewis lung carcinoma, metastasis, mice

## Abstract

The objective of this study was to determine whether a reduction in energy intake ameliorated the high-fat diet-enhanced spontaneous metastasis of Lewis lung carcinoma in mice. Male C57BL/6 mice were fed the AIN93G diet, a high-fat diet or a high-fat diet with a 5% restriction of the intake. Energy restriction reduced body adiposity and body weight, but maintained growth similar to mice fed the AIN93G diet. The high-fat diet significantly increased the number and size (cross-sectional area and volume) of metastases formed in lungs. Restricted feeding reduced the number of metastases by 23%, metastatic cross-sectional area by 32% and volume by 45% compared to the high-fat diet. The high-fat diet elevated plasma concentrations of proinflammatory cytokines (monocyte chemotactic protein-1, plasminogen activator inhibitor-1, leptin), angiogenic factors (vascular endothelial growth factor, tissue inhibitor of metalloproteinase-1) and insulin. Restricted feeding significantly reduced the high-fat diet-induced elevations in plasma concentrations of proinflammatory cytokines, angiogenic factors and insulin. These results demonstrated that a reduction in diet intake by 5% reduced high-fat diet-enhanced metastasis, which may be associated with the mitigation of adiposity and down-regulation of cancer-promoting proinflammatory cytokines and angiogenic factors.

## INTRODUCTION

Overweight and obesity affect cancer survival and are associated with increased mortality caused by cancer in the U.S. [1]. Recurrent and metastatic cancer remains the most devastating aspect of cancer. Obesity at the time of cancer diagnosis can be predictive of increased risk of early recurrence and metastasis [2–5]. Animal studies support the clinical observation that consumption of an obesogenic, high-fat diet increases primary tumorigenesis [6–8] and metastasis [6, 9].

Weight reduction through energy restriction is considered useful in alleviating obesity and obesity-associated cancer risk. Dietary energy restriction reduces body adiposity and body weight and improves energy metabolism [10, 11]. Furthermore, energy restriction results in favorable alterations of serum hormonal and biological factors that are related to increased risk for cancer recurrence in obese breast cancer survivors [12] and in overweight and obese women who are at increased risk of breast cancer [13]. Animal studies show that energy restriction is effective in reducing primary tumorigenesis in various models [14–16]. However, few studies have investigated the efficacy of energy restriction on metastasis.

We reported that feeding mice a high-fat diet enhances spontaneous metastasis of Lewis lung carcinoma (LLC) in lungs [17, 18]. We hypothesized that reduction in energy intake reduces high-fat diet-enhanced metastasis. The present study tested the hypothesis by using the LLC spontaneous metastasis model in which mice were fed a high-fat diet with a 5% reduction in intake. The rationale of choosing a 5% restriction was to maintain growth similar to mice fed the AIN93G control diet and to avoid possible growth retardation, which possibly could attenuate the host defense against malignant aggression.

## RESULTS

**Figure 1:**
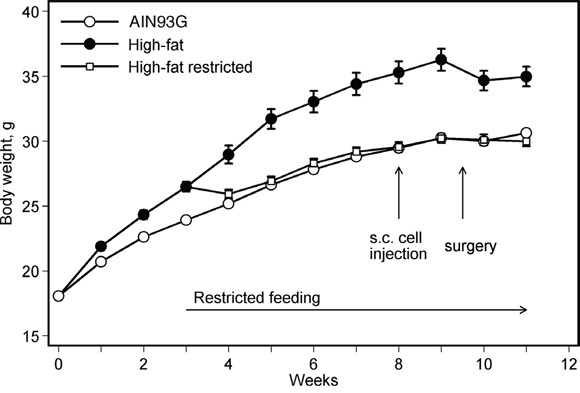
Restricted feeding reduces body weight in mice fed a high-fat diet Values are means ± SEM (*n* = 35-38 per group). Mice fed the high-fat diet were heavier than those fed the AIN93G diet; the difference was significant two weeks after the initiation of experimental feeding (*p* < 0.05). Restricting diet intake by 5% reduced body weight of mice fed the high-fat diet; the difference was significant one week after the initiation of restricted feeding (*p* < 0.05).

Unrestricted feeding of the high-fat diet increased body weight (Figure 1). The weight was different from that for mice fed the AIN93G diet two weeks after the initiation of the high-fat diet feeding (*p* < 0.05). The higher body weight was maintained throughout the experiment (Figure 1). Restricting the high-fat diet intake by 5% reduced body weight to levels similar to mice fed the AIN93G diet (Figure 1). The reduction was significant one week after the initiation of the restricted feeding (*p* < 0.05); the lower body weight was maintained throughout the experiment (Figure 1).

**Figure 2:**
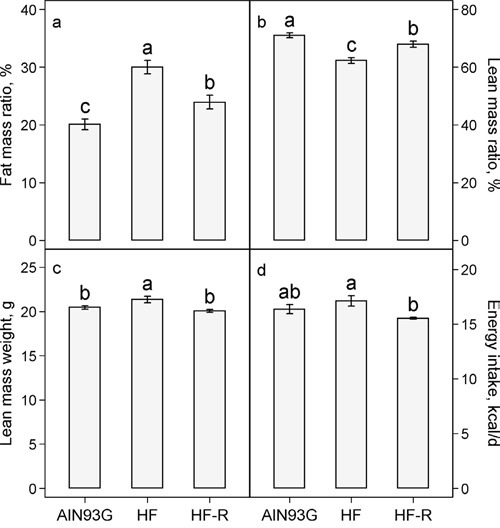
Effects of restricted feeding on a. fat mass: body mass ratio b. lean mass: body mass ratio c. lean mass weight and d. energy intake in mice fed a high-fat diet Values (means ± SEM) with different letters are significantly different at *p* < 0.05 (*n* = 35-38 per group, *n* = 6 for energy intake). AIN93G: AIN93G diet; HF: high-fat diet; HF-R: 5% restriction of the high-fat diet.

In groups receiving unrestricted feeding, the high-fat diet compared to the AIN93G diet increased the percent body fat mass by 50% (Figure 2a) and correspondingly reduced the percent lean body mass by 12% (Figure 2b). Restricted feeding of the high-fat diet reduced the body fat mass by 20% (Figure 2a) and increased the body lean mass by 9% (Figure 2b). Pearson correlation analysis showed that body weight was positively correlated with body fat mass weight (*r* = 0.91, *p* < 0.01). Unrestricted feeding of the high-fat diet elevated the lean mass weight by 4% (Figure 2c). The lean mass weight of the restricted group was 6% lower than that of the group fed the unrestricted high-fat diet, but it was similar to that of the AIN93G-fed group (Figure 2c). There was no significant difference in energy intake between groups fed the AIN93G and the high-fat diet (Figure 2d). Restricted feeding of the high-fat diet, compared to unrestricted, reduced energy intake by 9% (Figure 2d).

**Figure 3:**
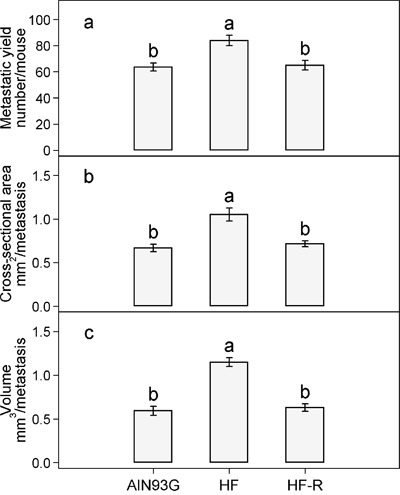
Restricted feeding reduces the a. number b. cross-sectional area and c. volume of lung metastases in mice fed the high-fat diet Values (means ± SEM) with different letters are significantly different at *p* < 0.05 (*n* = 34-36 per group). AIN93G: AIN93G diet; HF: high-fat diet; HF-R: 5% restriction of the high-fat diet.

Subcutaneous injection of LLC cells resulted in a primary tumor at the injection site and metastases in lungs. There was no significant difference in primary tumor weight among the three groups; the overall average was 0.34 ± 0.01 g/tumor. The number of lung metastases in mice fed the unrestricted high-fat diet was 32% higher than that in mice fed the AIN93G diet (Figure 3a). Restricted compared to unrestricted intake of the high-fat diet reduced the number of metastases by 23% (Figure 3a). Compared to the AIN93G diet, the high-fat diet increased metastatic cross-sectional area by 57% (Figure 3b) and volume by 94% (Figure 3c). Restricted compared to unrestricted intake of the high-fat diet reduced the cross-sectional area by 32% (Figure 3b) and the volume by 45% (Figure 3c).

**Figure 4:**
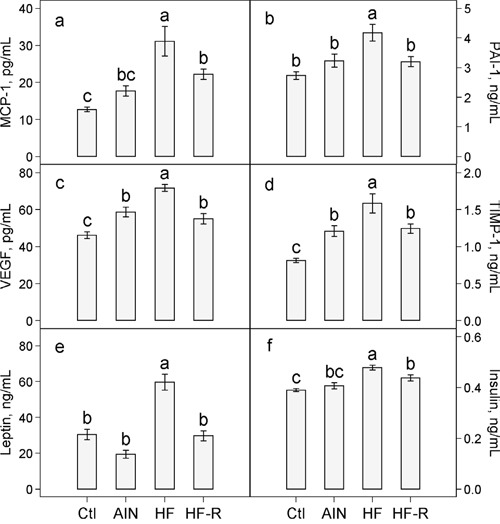
Effects of restricted feeding on plasma concentrations of a. MCP-1 b. PAI-1 c. VEGF d. TIMP-1 e. leptin and f. insulin in mice fed the high-fat diet Values (means ± SEM) with different letters are significantly different at *p* < 0.05 (*n* = 10 per group). Ctl: non-tumor-bearing mice fed the AIN93G diet; AIN: AIN93G diet; HF: High-fat diet; HF-R: 5% restriction of the high-fat diet.

There were no significant differences in plasma concentrations of MCP-1 (Figure 4a) and PAI-1 (Figure 4b) in AIN93G-fed mice with or without LLC. In LLC-bearing mice, unrestricted feeding of the high-fat diet increased plasma MCP-1 by 76% (Figure 4a) and PAI-1 by 29% (Figure 4b). Restricted compared to unrestricted feeding of the high-fat diet reduced MCP-1 by 29% (Figure 4a) and PAI-1 by 23% (Figure 4b).

Plasma concentrations of VEGF and TIMP-1 were 27% (Figure 4c) and 49% (Figure 4d) higher, respectively, in LLC-bearing mice than in non-tumor-bearing mice fed the AIN93G diet. Unrestricted feeding of the high-fat diet elevated plasma VEGF by 22% (Figure 4c) and TIMP-1 by 31% (Figure 4d) in LLC-bearing mice. Restricted compared to unrestricted feeding of the high-fat diet resulted in a 23% reduction in VEGF (Figure 4c) and a 22% reduction in TIMP-1 (Figure 4d).

There were no significant differences in plasma concentrations of leptin and insulin in AIN93G-fed mice with or without LLC (Figure 4e and 4f). In LLC-bearing mice, unrestricted feeding of the high-fat diet compared to the AIN93G diet increased plasma leptin by 3-fold (Figure 4e) and insulin by 18% (Figure 4f). Restricted compared to unrestricted feeding of the high-fat diet lowered plasma leptin by 50% (Figure 4e) and insulin by 9% (Figure 4f).

## DISCUSSION

Consistent with our previous reports [9, 17], the present study showed that feeding mice an obesogenic, high-fat diet enhances spontaneous metastasis of LLC in the lungs. A 5% reduction in intake of this diet reduced the number and size of metastases formed in the lungs, indicating that restricted feeding reduces high-fat diet-enhanced metastasis.

Reduction in pulmonary metastasis by restricted feeding is accompanied with reductions in body fat mass and concentrations of proinflammatory cytokines in plasma. Adipose tissue is an endocrine organ that produces proinflammatory cytokines. For example, feeding mice a high-fat diet significantly elevated concentrations of MCP-1 [19] and PAI-1 in adipose tissue [18]. Elevation in proinflammatory cytokines is associated with cancer progression [20, 21]. Knocking out MCP-1 [19] or PAI-1 genes from mice [18] reduces high-fat diet-enhanced metastasis. A reduction in body adiposity may lead to a decreased production of proinflammatory cytokines including MCP-1 and PAI-1, which may be responsible, at least partly, for the anti-metastatic effects of the restricted feeding.

Angiogenesis plays an important role in tumorigenesis and transporting metastatic cells to target organs. Both VEGF and TIMP-1 are potent angiogenic factors. We previously found that plasma concentrations of VEGF and TIMP-1 are elevated in mice with LLC metastases, and they are further elevated by feeding mice a high-fat diet [18, 19]. In the present study, the increases in plasma concentrations of VEGF and TIMP-1 with high-fat diet-enhanced metastasis indicate a stimulation of angiogenesis during LLC spread and growth. Restricted feeding of the high-fat diet significantly reduced concentrations of VEGF and TIMP-1, which suggests a down-regulation of angiogenesis. This down-regulation may contribute to the attenuation of LLC metastatic progression by the restricted feeding.

The lower concentrations of leptin and insulin in plasma of mice receiving restricted feeding were likely the result of reduced adiposity. Leptin and insulin actively participate in energy metabolism and their elevations in blood correlate with metabolic disturbance in rodent models of obesity [22, 23]. Furthermore, leptin is angiogenic during tumorigenesis [24], and insulin is involved in type-2 diabetes-mediated mammary tumor progression in mice [25]. Reductions in leptin and insulin indicate that restricted feeding may attenuate metabolic disturbance by intake of the high-fat diet, and such an action may contribute to the anti-metastatic effects of restricted feeding.

Energy restriction, ranging from 20% to 40%, has been used to induce weight loss in rodent models in cancer prevention research [15, 26–28]. Previously, we found that a 30% restriction of high-fat diet intake halted growth and a 20% restriction significantly retarded growth of C57BL/6 mice, while a 7% restriction resulted in a slightly lower but similar growth to mice fed the AIN93G diet (unpublished data). Thus, we chose to restrict the intake by 5% because food intake should not be reduced to the point where minimum energy needs for physiological growth and maintenance of animals cannot be met. Energy restriction is defined as a reduction in energy intake without malnutrition [29]. Growth retardation due to malnutrition because of energy deprivation should be distinguished from weight and adiposity loss due to energy restriction. This is particularly important in cancer prevention research. Malignant growth competes against the host for nutrients to support its rapid progression. Caution should be taken to avoid energy deprivation or insufficiency in models of weight loss and maintenance, which may bias the results and their interpretation.

In summary, results from this study showed that restricting the high-fat diet intake by 5%, which reduced body adiposity and body weight but maintained normal growth of mice, reduced high-fat diet-enhanced spontaneous metastasis. Inhibition of metastasis by restricted feeding is likely through mechanisms of rebalancing metabolic homeostasis by reducing adipogenesis and downregulating its associated production of cancer-promoting proinflammatory cytokines and angiogenic factors. Furthermore, it suggests that dietary energy restriction, by reducing body adiposity and maintaining a healthy body weight, may reduce the severity of occurrence and metastasis in overweight or obese cancer patients after treatment of primary cancer, and thus improve prognosis and quality of life.

## MATERIALS AND METHODS

### Animals and diets

Three-week-old male C57BL/6 mice (Harlan, Madison, WI) were maintained in a pathogen-free room with a 12:12-hour light/dark cycle and a temperature of 22 ± 1°C. Three diets were used in this study, the AIN93G diet [30] containing 16% or 45% (high-fat diet) of energy from corn oil, or the high-fat diet for the 5% restricted feeding in that the nutrient density was adjusted to be equivalent to that of the high-fat diet for the unrestricted feeding (Table 1). All diets were powder diets; they were stored at −20°C until feeding. Gross energy of each diet (Table 1) was analyzed by using oxygen bomb calorimetry (Model 6200, Oxygen Bomb Calorimeter, Parr Instrument, Moline, IL).

**Table 1:** Composition of diets

	AIN93G	High-fat	High-fat for 5% restricted feeding
Ingredient	g/kg	g/kg	g/kg
Corn Starch	397.5	40.2	22.6
Casein	200	239.4	252.5
Dextrin	132	239.4	239.4
Sucrose	100	119.7	119.7
Corn oil	70	241.1	242.5
Cellulose	50	59.8	59.8
AIN93 mineral mix	35	41.9	44.0
AIN93 vitamin mix	10	12	12.6
L-Cystine	3	3.6	3.8
Choline bitartrate	2.5	3	3.1
*t*-Butylhydroquinone	0.014	0.02	0.02
Total	1000	1000	1000
			
Energy	%	%	%
Protein	20	20	21.3
Fat	16	45	45.2
Carbohydrate	64	35	33.5
			
Gross energy kcal/g ^a^	4.4 ± 0.1	5.3 ± 0.1	5.3 ± 0.1

a Values are means ± SEM of five samples analyzed from each diet.

### Lewis lung carcinoma cells

Lewis lung carcinoma (LLC) cell line, a variant that metastasizes to lungs [31], was obtained from Dr. Pnina Brodt, McGill University, Montreal, Quebec, Canada. The cells were cultured with RPMI-1640 medium containing 10% heat-inactivated fetal bovine serum and maintained in a humidified atmosphere of 5% CO_2_ in air at 37°C. Cells used for animal studies were *in vivo*-selected once [9]. The cells were monitored for phenotype by microscopic examination of cell morphology, proliferation properties by growth curve analysis and metastatic capability by injecting cells subcutaneously into mice and examining metastatic formation in lungs. Cells were free of mycoplasma based on Hoechst DNA staining and direct culture tests (performed by American Type Cell Collection, Manassas, VA). These assessments showed that cell identity and metastatic behavior were similar to those of original stocks from the institution providing the cell line.

### Experimental design

This study was approved by the Institutional Animal Care and Use Committee of the U.S. Department of Agriculture, Agricultural Research Service, Grand Forks Human Nutrition Research Center. The procedures followed the National Institutes of Health guidelines for the care and use of laboratory animals [32].

After acclimation with the AIN93G diet for one week, mice were randomly assigned into two groups and fed the AIN93G (*n* = 36) and the high-fat diet (*n* = 73), respectively. Food intake measurements (*n* = 6 per group) were initiated two weeks later when significant differences in body weight occurred between the two groups. At week three, mice fed the high-fat diet were divided into two groups; one remained on unrestricted access to the diet (*n* = 38), and the other was fed 95% of the amount that the unrestricted group consumed in the previous day (*n* = 35). To avoid food loss by spilling, diet was provided to the restricted group twice daily, one half at 8:00 a.m. and the other half at 4:00 p.m. Body composition was assessed in conscious, immobilized mice one week before cancer cell injection by using quantitative magnetic resonance imaging (Echo whole-body composition analyzer, Model 100, Echo Medical System, Houston, TX). Five weeks after the initiation of the restricted feeding, mice were subcutaneously injected with 2.5 × 10^5^ viable LLC cells per mouse into the lower dorsal region. The resulting subcutaneous tumor was resected surgically 10 days later when it was approximately one cm in diameter. Following surgery, mice were maintained on their respective diets for an additional 10 days. Mice fed the AIN93G diet but not injected with cancer cells served as controls to assess changes in plasma concentrations of cytokines and related biomarkers due to metastasis in LLC-bearing mice fed the AIN93G diet. Mice with recurrence after surgery were excluded from the study.

At termination, mice were intraperitoneally injected with a mixture of ketamine/xylazine. Lungs were harvested and fixed with Bouin's solution. The number of pulmonary metastases was counted [33] and the cross-sectional area and the volume of each metastasis were analyzed [34] by using a camera-equipped stereomicroscope and ImagePro-Plus software (Media Cybernetics, Silver Spring, MD). The cross-sectional area of a metastasis was defined as the surface area of the lung metastasis. The volume was estimated by assuming that the metastasis was spherical and using its average diameter [34]. The average diameter was the average measured at two degree intervals joining two outline points and passing through the centroid. Plasma was collected and stored at −80°C for quantifying proinflammatory cytokines, angiogenic factors and insulin.

### Concentrations of cytokines, angiogenic factors and insulin in plasma

Sandwich enzyme-linked immunosorbent assay (ELISA) kits were used to quantify plasma concentrations of proinflammatory cytokines (leptin, monocyte chemotactic protein-1 (MCP-1) and plasminogen activator inhibitor-1 (PAI-1)), angiogenic factors (vascular endothelial growth factor (VEGF) and tissue inhibitor of metalloproteinase-1 (TIMP-1)) and insulin following manufacturers' protocols. Leptin, MCP-1, PAI-1, VEGF and TIMP-1 ELISA kits were obtained from R&D Systems (Minneapolis, MN), and the insulin kit was from Mercodia (Winston-Salem, NC). Samples were read within the linear range of the assay, and the accuracy of the analysis was confirmed by the controls provided in each kit.

### Statistical analyses

One-way analysis of variance (ANOVA) and Tukey contrasts were used to compare differences among the groups. Pearson correlation was performed between body weight and body fat mass weight. A mixed model ANOVA with mouse as the random blocking factor was used to compare differences in size of metastases (cross-sectional area and volume) in mice fed different diets. All data are presented as means ± standard error of the mean (SEM). Differences with a *p* value of 0.05 or less are considered significant. All analyses were performed by using SAS software (version 9.4, SAS Institute, Cary, NC).
